# Universal CRISPR/Cas12a-associated aptasensor suitable for rapid detection of small proteins with a plate reader

**DOI:** 10.3389/fbioe.2023.1201175

**Published:** 2023-06-02

**Authors:** Yi Li, Linyang Liu, Laicong Qiao, Fei Deng

**Affiliations:** ^1^ Graduate School of Biomedical Engineering, Faculty of Engineering, University of New South Wales, Sydney, NSW, Australia; ^2^ ARC Centre of Excellence for Nanoscale Biophotonics, University of New South Wales, Sydney, NSW, Australia

**Keywords:** CRISPR/Cas12a, aptamer, universal, cytokines, small molecule

## Abstract

With the discovery of the collateral cleavage activity, CRISPR/Cas12a has recently been identified as a key enabling approach in novel DNA biosensor development. Despite its remarkable success in nucleic acid detection, realizing a universal CRISPR/Cas biosensing system for non-nucleic acid targets remains challenging, particularly at extremely high sensitivity ranges for analyte concentrations lower than the pM level. DNA aptamers can be designed to bind to a range of specific target molecules, such as proteins, small molecules, and cells, with high affinity and specificity through configuration changes. Here, by harnessing its diverse analyte-binding ability and also redirecting the specific DNA-cutting activity of Cas12a to selected aptamers, a simple, sensitive, and universal biosensing platform has been established, termed CRISPR/Cas and aptamer-mediated extra-sensitive assay (CAMERA). With simple modifications to the aptamer and guiding RNA of Cas12a RNP, CAMERA demonstrated 100 fM sensitivity for targeting small proteins, such as IFN-γ and insulin, with less than 1.5-h detection time. Compared with the gold-standard ELISA, CAMERA achieved higher sensitivity and a shorter detection time while retaining ELISA’s simple setup. By replacing the antibody with an aptamer, CAMERA also achieved improved thermal stability, allowing to eliminate the requirement for cold storage. CAMERA shows potential to be used as a replacement for conventional ELISA for a variety of diagnostics but with no significant changes for the experimental setup.

## 1 Introduction

A unique programmable nuclease activity-induced *trans*-cleavage in certain Cas proteins, such as from the Cas12 and Cas13 families, paved a new way to develop CRISPR/Cas-assisted novel biosensing systems (Li et al., 2019; van Dongen et al., 2020). The successfully demonstrated CRISPR/Cas-based DNA/RNA detection systems, such as Cas13a-based SHERLOCK/SHERLOCKv2 (Gootenberg et al., 2017; Gootenberg et al., 2018), and Cas12a-based DETECTR and HOLMES (Li et al., 2018a; Chen et al., 2018), exhibit remarkable sensitivity, reaching the aM level (Li et al., 2019; Aman et al., 2020). Cas nuclease, in these CRISPR/Cas-assisted nucleic acid biosensing systems, is guided by a specifically designed short RNA, termed guiding RNA (gRNA) or transcript RNA (crRNA), to recognize and bind to its complementary DNA or RNA sequence. The presence of a complementary nucleic acid sequence to gRNA or crRNA activates its sequence-specific nuclease activity accompanied by promiscuous nuclease activity, which then indiscriminately cuts the surrounding single-stranded nucleic acids over multiple turnovers (Gootenberg et al., 2017; Li et al., 2018b; Chen et al., 2018). With the introduction of properly designed single-stranded nucleic acid reporters, such unique dual-mode activation of the CRISPR/Cas RNP complexes is capable of realizing integrated target recognition and amplification functions. Subsequentially, such sequence-triggered collateral cleavage activity was successfully combined by other authors with additional recognition elements, such as the allosteric transcription factor (aTF) or aptamer, for realizing non-nucleic acid target detection, including proteins, small molecules, and ions. (Dai et al., 2020; Zhang et al., 2021). Although the remarkable flexibility and versatility of CRISPR/Cas RNPs combined with different biosensing components and materials indicate a bright future for integration of CRISPR/Cas in various biosensing systems (Li et al., 2021a; Li et al., 2022), the simplicity and universality of such systems for non-nucleic acid detection remain challenging.

Based on the conformational changes of the nucleic acid structure, aptamers, a unique type of specifically selected nucleic acid oligos, exhibit superior binding affinity to interact with specific analytes (Seo and Gu, 2017; Munzar et al., 2019). In the past two decades, with their exceptional compatibility and versatility compared to other recognition elements, aptamers have been widely applied for biosensor development to detect a variety of analytes, such as proteins (Dehghani et al., 2018), ions (heavy metal) (McConnell et al., 2020), antibiotics (Mehlhorn et al., 2018), small molecules (Nguyen et al., 2017), pathogens (Li et al., 2021b), and cancer biomarkers (Zhou et al., 2014), and they have been recognized as a potential substitute for the most widely used conventional antibodies for non-nucleic acid detection (Zhou et al., 2014; Nguyen et al., 2017; Preuss et al., 2020). The integration of aptamers with a variety of different nanomaterials and signal generation strategies has been successfully demonstrated in diverse biosensor system developments (Song et al., 2008; Han et al., 2010). Recently, aptamers have also been applied in combination with the Cas12a effector for non-nucleic acid analyte detection (Deng et al., 2022). Based on the activation efficiency changes of Cas12a to the aptamer sequence with or without the binding of its designated target analyte, a Cas12a-based electrochemical biosensing system has been demonstrated to detect TGF- β1, a small protein, with 10 nM sensitivity (Dai et al., 2019). A similar strategy has also been used for detecting ATP by Peng et al. (2020) in a design which yielded 400-nM sensitivity along with a linear range from 1 μM to 200 µM. In addition, other types of aptamers, including the aptamer-based allosteric hairpin probes, such as DNAzymes (Li et al., 2016; Wang et al., 2019; Wang et al., 2020), have also been used to link CRISPR/Cas12a with non-nucleic acid analyte detection. Examples include the ASD-Cpf1 system reported by Wang et al. (2020) for ATP detection with 1.8-pM LOD with a linear range from 2 pM to 10 µM, and the DNAzyme-induced Cas12a *trans*-cleavage for detection of Pb^2+^ and pathogens with LOD reaching ∼53 pM and ∼3 CFU/mL, respectively (Li et al., 2020). However, the majority of aptamer-based biosensors have shown limitations in sensitivity between nM to µM ranges and a relatively narrow linear detection range of 2–3 orders of magnitude (Seok Kim et al., 2016; Peng et al., 2020). Such biosensing performance may be insufficient for certain diagnostic scenarios, such as for cytokine diagnostics or for certain biomarkers, where sub-pM concentration limits and wider dynamic ranges may be needed (Chikkaveeraiah et al., 2012; Wu et al., 2019).

In this study, a simple but universal novel biosensing platform has been established by combining a dsDNA-based aptamer sensor with CRISPR/Cas12a-based fluorescence signal amplification. We termed our platform the CRISPR/Cas and aptamer-mediated extra-sensitive analyzer (CAMERA). Taking advantage of aptamers being nucleic acid oligonucleotides, their diverse and highly specific non-nucleic acid target binding affinity can act as a bridge between molecularly specific detection with the dual-mode nucleic acid cleavage function of CRISPR/Cas12a for enhancing the overall detection sensitivity. Our CAMERA has successfully demonstrated its capability to quantitatively detect small proteins, such as cytokine IFN-γ, with a sensitivity of 100 fg/mL (fM level) with five orders of magnitude linear range from 100 fg/mL to 10 ng/mL. In addition, IFN-γ detection from plasma samples has also been demonstrated using a simple dilution process during sample preparation. In these conditions, CAMERA has been able to reach a sensitivity of 8 pg/mL with a linear correlation from 8 pg/mL to 5 ng/mL. An additional analyte, insulin, has also been used for testing the universality of CAMERA. By simply changing the aptamer sequence and the gRNA sequence of Cas12a RNP, CAMERA was redirected to target insulin, where it exhibited the same 100 fg/mL sensitivity and five orders of magnitude linear range. Notably, our CAMERA is built on a standard 96-well plate, and it uses an ELISA-like procedure but with a significantly short detection time (∼1.5 h) compared to a general ELISA kit (3–4 h). Such a standard 96-well plate-based CAMERA represents a user-friendly alternative approach that is potentially capable of being applied to antibody-hostile diagnostic scenarios and can be easily accommodated in existing ELISA diagnostic facilities without additional problems of instruments and cost.

## 2 Materials and methods

### 2.1 Chemicals and materials

The following materials were used in the study: PBS (Sigma), BSA (Sigma), agarose (Sigma), 10,000X SYBG Gold nucleic acid dye (Thermo Fisher), a high-binding 96-well plate, 10-bp DNA ladder (Thermo Fisher), Cas12a protein (Cpf1, NEBiolabs), NEBuffer 2.1 (10X, NEBiolabs), streptavidin (Sigma), 6X DNA loading dye (Thermo Fisher), IFN-γ (R&D), IL-1β (R&D), IL-6 (R&D), TNF-α (R&D), IL-10 (R&D), IL-2 (R&D), insulin (Sigma), glucose (Glu, Sigma), sucrose (Sur, Sigma), ascorbic acid (AA, Sigma), uric acid (UA, Sigma), and IgG.

All plasma-related works were covered under UNSW Ethics Approval HC210160.

All DNA and RNA oligos were artificially synthesized by Sangon Biotech Co. Ltd., as shown in Supplementary Table S1.

### 2.2 Fluorescence-quenched assay for dsDNA formation

A total of 500 µL 1 µM aptamer ssDNA oligo was first mixed with 400 µL of 1 µM corresponding complementary DNA (cDNA) oligo (with or without BHQ2 and/or biotin labeling) in the 1X PBS solution. After vigorous vortexing for 10 s, the mixture was incubated at room temperature for different time periods (5, 10, 30, 60, 90, and 120 min, or overnight). Then, the fluorescence signal changes before and after mixing were measured using an iD5 plate reader at Ex = 570 nm/Em = 615 nm.

### 2.3 EMSA test to verify dsDNA formation

The EMSA was also performed to confirm the successful formation of the dsDNA structure between the aptamer and its cDNA: each 2 µL 6X loading dye was mixed with 10 µL of 1 µM aptamer oligo, 10 µL of 1 µM cDNA, and 10 µL of 1 µM pre-made dsDNA mixture. Then, these loading dye mixtures were loaded onto 2% agarose gel (SYBR Gold fluorescent dye pre-loaded). Afterward, a 10-bp DNA ladder was applied, and constant-voltage electrophoresis was conducted at 120 V for 1.5 h. Gel picture was captured by using the gel imaging platform (Bio-Rad, Doc + XR) with a default setup for the SYBR dye.

### 2.4 Verification of the aptamer-based dsDNA response to the analyte

A measure of 100 µL of the 1-µM pre-made IFN-γ dsDNA biosensor, with the 5′-Texas Red-labeled aptamer and 3′-BHQ2-labeled cDNA, was mixed with 20 µL of IFN-γ PBS solution at different IFN-γ concentrations (26, 52.1, 104.2, 125, 208.3, 250, 416.6, 500 ng/mL and 1, 2 μg/mL). Then, 20 μL of a PBS solution with no IFN-γ was used as a control. After incubation in the dark at 37°C for 60 min (for continuous tests, the reaction mixtures were kept in the dark between readings), the fluorescence intensity was detected using a SpectraMax iD5 microplate reader with a setup of Ex = 570 nm/Em = 615 nm.

### 2.5 Aptamer-triggered Cas12a RNP collateral cleavage for signal amplification

First, the CRISPR/Cas12a signal amplification mixture was prepared as follows: 2.5 µL of 20 µM gRNA was gently mixed with 10 µL of 10 µM Cas12a nuclease in 5 mL 1X NEBuffer 2.1 (10 X NEBuffer 2.1 with DNase/RNase free water dilution). Afterward, 10 µL of the 100 µM fluorescence-quenched ssDNA reporter (5′-Texas Red, 3′-BHQ2) was gently mixed to form the CRISPR/Cas12a amplification mixture. The pre-made amplification mixture was then stored at 4°C for further use. Then, to investigate the CRISPR/Cas12a activation efficiency, 5 µL of 500 nM pre-made dsDNA or aptamer ssDNA was added to 100 µL of the pre-made amplification mixture. The reaction was set at RT for 1 h, followed by fluorescent intensity measurement using an iD5 microplate reader with a setup of Ex = 570 nm/Em = 615 nm. Alternatively, dynamic changes in the fluorescence signal were recorded by measuring the fluorescence intensity at 10-min intervals for 1 h. For evaluation of the CRISPR/Cas12a activation efficiency changes in the presence of the analyte, 100 µL of the pre-made CRISPR/Cas12a signal amplification mixture was mixed with 20 µL of 500 nM aptamer/dsDNA with the pre-mixed target analyte at 100 ng/mL final concentration. After RT incubation for 60 min, the fluorescence intensity was recorded using an iD5 microplate reader with a setup of Ex = 570 nm/Em = 615 nm.

### 2.6 Optimization of the Cas12a to gRNA ratio in signal amplification

In order to examine the optimal condition of CRISPR/Cas12a RNP for signal amplification, guide RNA solutions at different final concentrations (0, 1, 10, 25, 50, and 100 nM) were used to prepare the amplification mixture with a fixed Cas12a protein to a final concentration of 0.5 µM. Subsequently, different ratios of Cas12a protein to guide RNA were also tested for fluorescence signal generation. With a fixed Cas12a protein concentration at 0.5 µM, gRNA was added with molar ratios (Cas12a: gRNA) of 100:1, 10:1, 4:1, 2:1, and 1:1 to form the reaction mixtures. Afterward, 5 µL of the 1 µM aptamer solution with a complementary sequence to gRNA was added to 100 µL of each of the two types of pre-made CRISPR/Cas12a amplification mixtures, respectively. After 1 h incubation at RT, the fluorescence intensity was recorded using an iD5 microplate reader with a setup of Ex = 570 nm/Em = 615 nm.

### 2.7 Attaching the dsDNA biosensing module to a 96-well plate

A measure of 100 µL of 10 μg/mL streptavidin was first applied to a flat-bottomed polystyrene 96-well plate (high-binding) for 2 h at 4°C. Then, 300 µL of the 1X PBS solution was used to wash the wells twice, followed by adding 300 µL of 1 mg/mL BSA to block the wells at RT for 60 min. Afterward, 100 µL of various concentrations of pre-made dsDNA with biotin-labeled cDNA and the Texas Red-labeled aptamer (0, 0.1, 0.5, 1, 2.5, 5, 10, and 25 nM) was added and incubated at RT for 90 min for fixation. Followed by washing two times with 300 µL of 1X PBS (0.5 mg/mL BSA added), the dsDNA biosensing interface was formed. The signal from the fixed dsDNA was then detected using an iD5 microplate reader with a setup of Ex = 570 nm/Em = 615 nm or fluorescent microscopy with Ex = 600 nm. To verify the release of the aptamer from the dsDNA biosensing interface, 100 µL of the 50 pg/mL analyte was added to the plate and set at RT for 60 min. After washing three times with 300 µL of 1X PBS, 100 µL of 1X PBS was added, and the final signal intensity was measured using an iD5 microplate reader with a setup of Ex = 570 nm/Em = 615 nm.

### 2.8 Integrated CAMERA system for target detection

To perform target detection using our integrated CAMERA system, 100 µL of samples with different concentrations of IFN-γ (0, 0.1, 1, 10, 100 pg/mL and 1, 10 ng/mL) spiked in 1X PBS were, respectively, added onto the pre-made biosensing interface of a 96-well plate (dsDNA biosensing module without fluorophore labeling). After incubation at RT for 60 min, 300 µL of 1X PBS was used to wash the plate three times. Then, 100 µL of the pre-made CRISPR/Cas12a signal amplification solution was added to each well and incubated at RT for 30 min. Subsequently, the amplified fluorescent intensity was measured using an iD5 microplate reader with a setup of Ex = 570 nm/Em = 615 nm. To detect the plasma sample, the plasma was first diluted at 1X PBS to a final concentration of 100%, 80%, 60%, 40%, 20%, and 10%, and then, IFN-γ was spiked in at different concentrations. Then, the sample protocol was applied to this 100 µL of diluted and spiked plasma to perform the detection. For the detection of an additional analyte, insulin, the same dsDNA fixation was performed by using the insulin aptamer and its corresponding cDNA, respectively, along with the CRISPR/Cas12a signal amplification mixture prepared by gRNAs with a complementary sequence to insulin aptamer. Then, the same detection protocol was followed for the final signal detection.

### 2.9 CAMERA specificity test

The system specificity of CAMERA was also evaluated by applying different interference molecules. For IFN-γ detection, we compared the signals from 100 µL of 1 ng/mL IFN-γ with 100 µL of the 10 ng/mL solution of each interference (in 1X PBS buffer with 100 μg/mL BSA), including IL-1β, IL-6, TNF-α, IL-10, and IL-2. Then, the detection protocol described in Method 3.2.8 was followed to acquire the final fluorescence signal. Additionally, for testing the insulin system specificity, 100 µL of the 1 ng/mL insulin solution was compared to 100 µL of 10 ng/mL interferences of UA, AA, Glu, and Sur, all in 100 μg/mL BSA. The same CAMERA protocol, as described in Method 2.8, was followed to acquire the final fluorescence signal.

## 3 Results

### 3.1 Principle of the CAMERA biosensing platform

**FIGURE 1:**
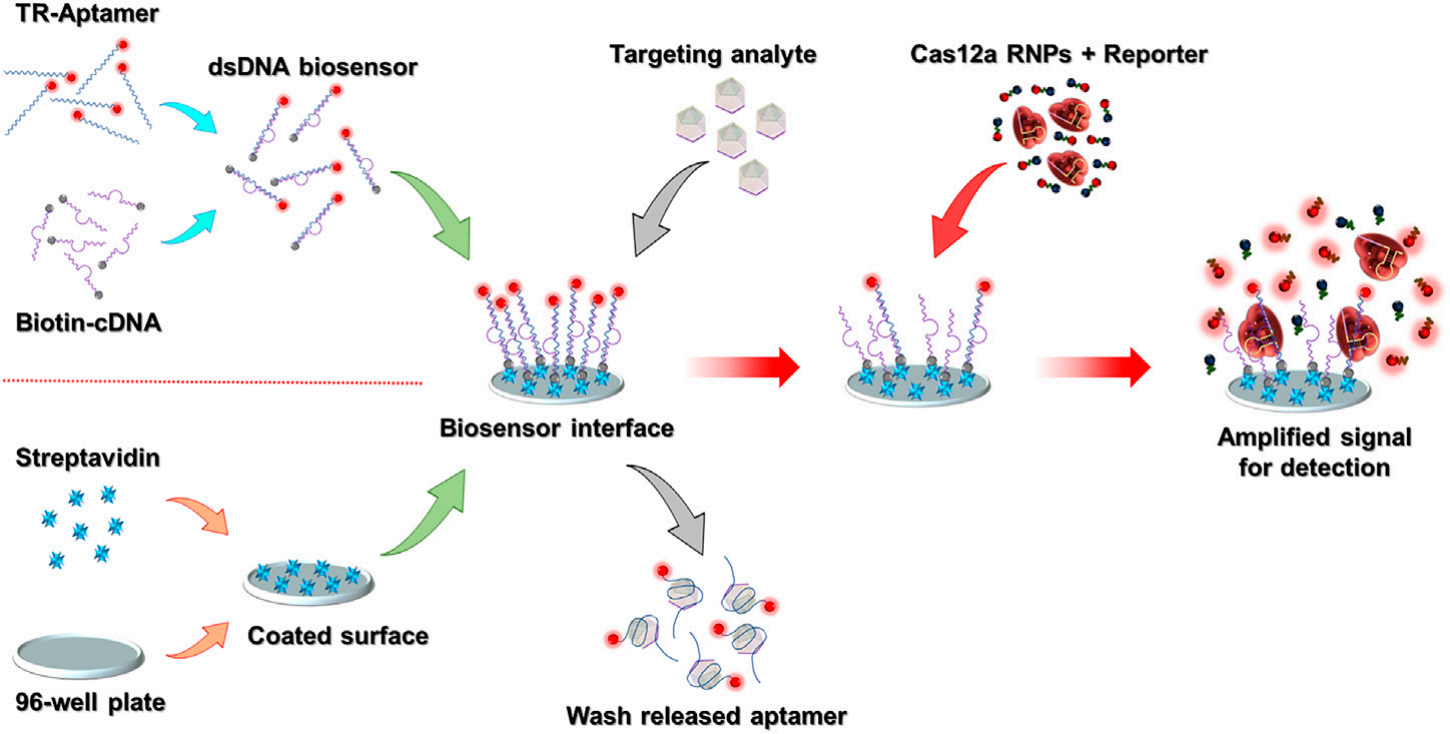
Schematic representation of the CAMERA platform. After the 96-well plate-based biosensing interface has been prepared, the whole detection process is conducted in three simple steps within ∼1.5 h: adding the sample, Cas12a signal amplification, and signal detection. The TR-aptamer was only used to demonstrate the function of the dsDNA biosensing module. In the integrated CAMERA platform, the aptamer does not require fluorophore labeling.

The CAMERA system is built on a standard high-binding 96-well plate, and the final signal can be directly detected by using any conventional ELISA plate reader with a fluorescence detection function. The whole system comprises an aptamer-based analyte recognition module and a CRISPR/Cas12a-based fluorescence amplification module. The schematic representation of the CAMERA platform is shown in Figure 1. In our implementation, a standard 96-well plate with high-binding polystyrene fabrication was coated with streptavidin. Simultaneously, the analyte recognition molecule was prepared by forming a dsDNA biosensor structure through mixing of the fluorescence-labeled ssDNA aptamer with biotinylated cDNA. Then, the dsDNA biosensor was fixed onto the streptavidin-coated 96-well plate surface through streptavidin-biotin binding to form the analyte recognition module. Within the presence of the target analyte, the aptamer tends to dissociate from the dsDNA structure and bind to the analyte because of its higher binding affinity to the designated target. Hence, the formed aptamer–analyte complex is released and then removed from the biosensing interface. In the next step, CRISPR/Cas12a RNPs and the quenched fluorescent reporters are added. The aptamer remaining on the sensing interface then activates the CRISPR/Cas12a RNPs and leads to a continuous uncontrolled cleavage of the reporters, separating the fluorophores from the quenchers and producing an amplified fluorescent signal. The magnitude of this signal correlates with the amount of the remaining aptamer. As higher quantities of the analyte presented to the surface lead to less aptamer–cDNA dsDNA remaining on the biosensing interface, the final fluorescence intensity from CRISPR/Cas12a amplification is negatively correlated with the concentration of the analyte.

### 3.2 Fabrication of the aptamer-based dsDNA biosensing module

**FIGURE 2:**
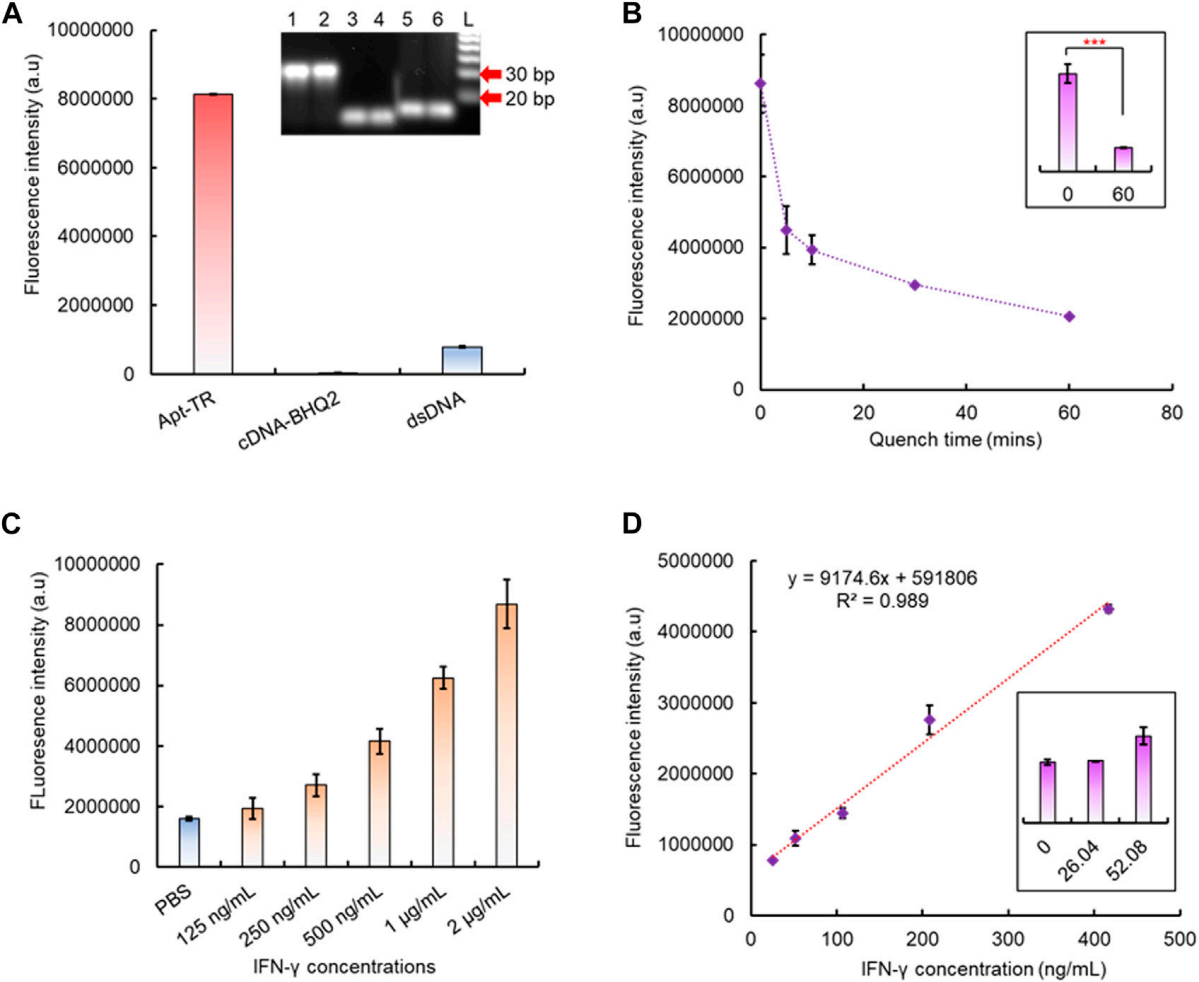
Fabrication of the dsDNA biosensor module. **(A)** For the fluorescence quenching test, a significantly decreased fluorescent signal between the formed dsDNA and free aptamer with Texas Red labeling (Apt-TR) indicated the successful formation of dsDNA. For gel electrophoresis, the image also confirmed the successful formation of the dsDNA structure. Due to the binding of the aptamer to its cDNA, the formed dsDNA molecule (line 1, 2) can be identified in the gel due to a significantly increased molecular weight in comparison to the free aptamer (lines 5, 6) or free cDNA (lines 3, 4). **(B)** Fluorescent signal changes during dsDNA formation. **(C)** IFN-γ aptamer-based dsDNA biosensor response to different analyte concentrations. **(D)** Linear correlation between the IFN-γ concentration and recovered fluorescent signal.

To verify the formation of the designed dsDNA structure, the IFN-γ aptamer with 5′ Texas Red fluorophore labeling and its cDNA with 3′ BHQ2 quencher labeling were mixed. In comparison to the solution with only the free aptamer with 5′ fluorophore labeling, the mixture of aptamer and cDNA exhibits a significant decrease in fluorescence signal intensity (Figure 2A), which indicates the formation of the dsDNA structure as the binding of cDNA to the aptamer leads to a close contact of the 5’ Texas Red fluorophore and the quencher. An agarose gel electrophoresis test also confirmed the successful dsDNA synthesis as the binding of cDNA to the aptamer caused their significantly delayed movement on the agarose gel compared to the free aptamer or ssDNA oligos, which led to the formation of bands with different molecule weight scales on the gel (Figure 2A). The formation of dsDNA between the aptamer and cDNA is a relatively quick process as the fluorescence signal decreased rapidly within the first 30 min after mixing (Figure 2B). As the quencher is labeled on the cDNA, the ratio of the aptamer to cDNA during mixing affects the quenching efficiency. A 10:5 ratio led to >80% quenching of the fluorescence signal, and increasing the ratio to 10:9 resulted in >95% quenching efficiency (Supplementary Figure S1). The quenching effect continued after 30 min as the fluorescence signal decreased slowly and continuously, but it eventually stabilized after 60 min (Supplementary Figure S2). Hence, the formed dsDNA structure was stable and non-reversible at room temperature, and 60 min was then selected as the time to prepare the dsDNA during the following tests. Afterward, a competitive binding test was performed to identify the binding affinity of the IFN-γ aptamer between IFN-γ and cDNA. The results have shown that in the presence of the target analyte IFN-γ, the dsDNA formation-induced fluorescence quenching effect has been obviously reduced (Supplementary Figure S3A), and the maximum difference was observed at 60 min reaction time (Supplementary Figure S3B).

After the dsDNA biosensor was formed, its target recognition function, in response to the target analyte, was also verified. With addition of different concentrations of IFN-γ (0, 0.125, 0.25, 0.5, 1, and 2 μg/mL) into the prepared aptamer–cDNA dsDNA biosensor solution, the increase of the fluorescence signal showed the successful aptamer–analyte binding (here, IFN-γ) and its release from the fluorescence-quenched dsDNA structure. Higher concentrations of the analyte result in the generation of higher fluorescence intensity as more dsDNA are opened by IFN-γ (Figure 2C). With increased reaction time, the fluorescence signal also increased and reached saturation at 60 min (Supplementary Figure S4). Notably, for this solution-based biosensor, the ratio between the aptamer and cDNA can significantly affect the fluorescence intensity, and the results have shown that a 10:8 ratio between the aptamer and cDNA yields the highest increase of fluorescence intensity in response to the analyte (Supplementary Figure S5). Applying the optimized 10:8 aptamer to the cDNA ratio in the IFN-γ dsDNA biosensor, a positive linear correlation was observed for responding to different concentrations of IFN-γ between 26 ng/mL and 400 ng/mL. The detection limit was found to reach 52.1 ng/mL (Figure 2D).

### 3.3 Demonstration of aptamer-triggered CRISPR/Cas12a collateral cleavage

**FIGURE 3:**
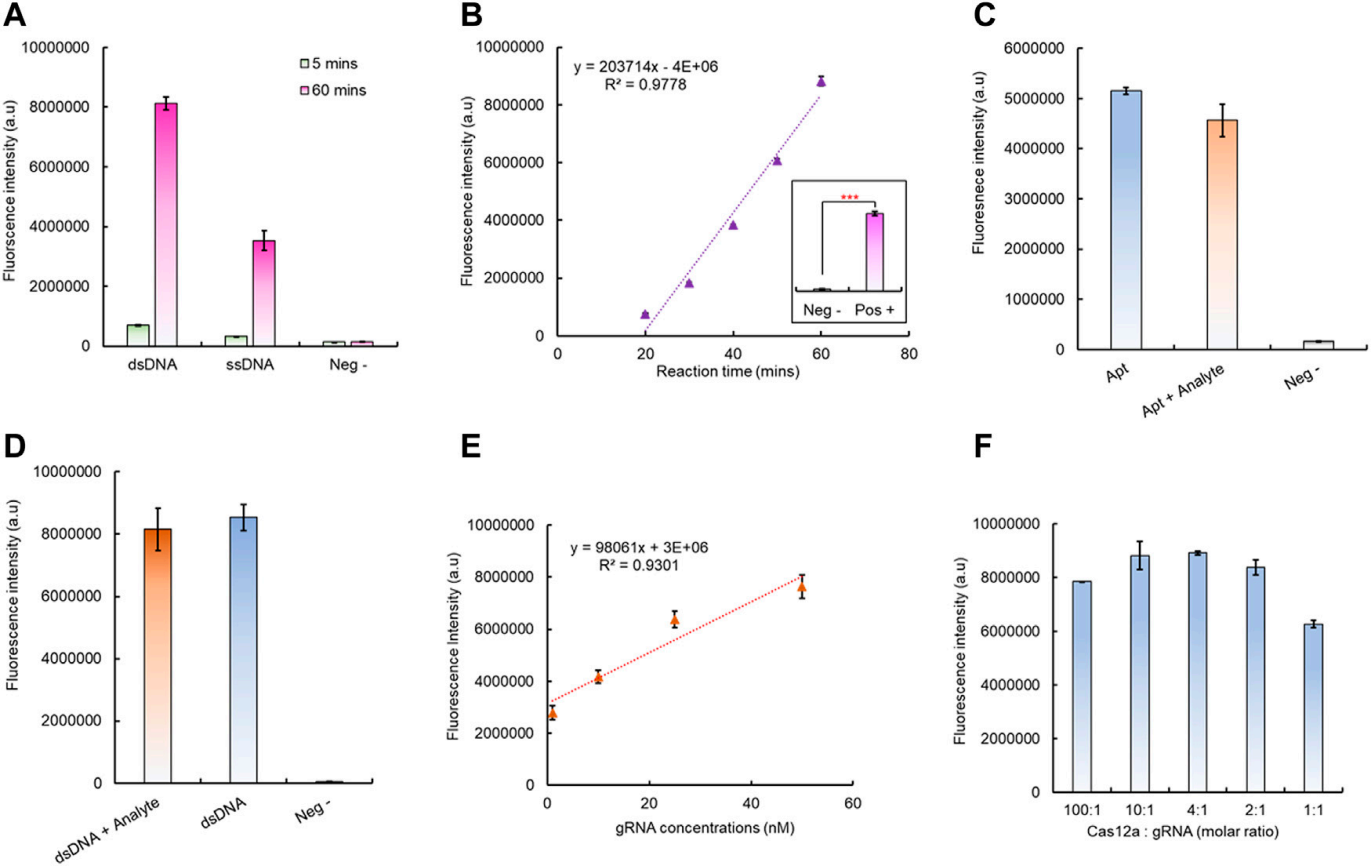
Verification of the aptamer-induced CRISPR/Cas12a collateral cleavage for signal amplification. **(A)** Aptamer-triggered Cas12a collateral cleavage activity and the Cas12a activation efficiency between different forms of the aptamer. **(B)** Cas12a collateral cleavage has a linear correlation between reaction time and the detected fluorescent signal. **(C)** Testing the Cas12a triggering efficiency changes between the free aptamer and aptamer/analyte mixture. **(D)** Testing the as12a triggering efficiency changes between the dsDNA biosensor module with/without the analyte. **(E)** Linear correlation between the gRNA concentration and Cas12a RNP trans-cleavage intensity, with 1 h reaction time. **(F)** Optimization of the gRNA to Cas12a ratio for signal amplification, with 1 h reaction time.

After successful fabrication of the aptamer-based dsDNA biosensor, its ability to trigger the Cas12a RNP was tested; this is critical for the integration of downstream CRISPR/Cas12a fluorescence signal amplification. To verify this, both the aptamer and dsDNA (without Texas Red and BHQ2) have been added to the CRISPR/Cas12a reaction mixture with a gRNA sequence complementary to the aptamer ssDNA sequence. The results have shown that both the dsDNA biosensor and ssDNA aptamer led to a significant increase of fluorescence intensity after 60 min at room temperature (Figure 3A), which indicated that Cas12a RNP was successfully activated by the aptamer sequence, either in ssDNA or dsDNA form. Compared to the aptamer alone, the dsDNA formation led to a higher collateral cleavage efficiency (Figure 3A), which is in agreement with previously reported studies (Li et al., 2018b; Chen et al., 2018). The activated CRISPR/Cas12a RNP can continuously cut its surrounding reporters; hence, it has led to accelerated fluorescence signal amplification with increasing reaction time and has shown a positive linear correlation between 20 min and 60 min (Figure 3B).

In the presence of an analyte, the aptamer tends to bind to the analyte. Therefore, the change of CRISPR/Cas12 triggering efficiency after the aptamer binding to its target analyte has also been evaluated. Compared to the aptamer alone for CRISPR/Cas12a RNP activation, the fluorescence intensity has shown no significant decrease for the aptamer mixed with the analyte before introducing CRISPR/Cas12a for signal amplification (Figure 3C). A similar situation was also observed for dsDNA with the analyte for CRISPR/Cas12a activation as no significant signal changes were identified, and both can continuously generate amplified fluorescence signals with increasing reaction time (Figure 3D, Supplementary Figure S6). Hence, CRISPR/Cas12a can recognize its target aptamer sequence and activate the collateral cleavage without being affected by the different configuration statuses of the aptamer sequence.

Notably, the change in the gRNA concentration can also affect the CRISPR/Cas12a collateral cleavage efficiency. The results indicated that, from 1 nM to 50 nM, the higher gRNA concentration led to a higher final fluorescence intensity, and with the increase in the gRNA concentration, a linear correlation between the gRNA concentration and final fluorescence intensity can be observed (Figure 3E, Supplementary Figure S7). However, the optimal concentration of gRNA depends on the molar ratio between Cas12a protein and the gRNA, and both 10:1 and 4:1 M ratios yield the same highest level of fluorescence intensity within the shortest time (Figure 3F, Supplementary Figure S8). Here, considering that RNA is relatively unstable before forming Cas12a RNP, a 4:1 M ratio has been selected for all following tests. Additionally, the stability of the prepared CRISPR/Cas12a reaction mixture has also been investigated. The Cas12a RNP activity level started to drop significantly after 3 days storage at 4°C, and >80% activity was still remaining for up to 10 days (Supplementary Figure S9).

### 3.4 Fixation of the dsDNA biosensing module onto a 96-well plate

**FIGURE 4:**
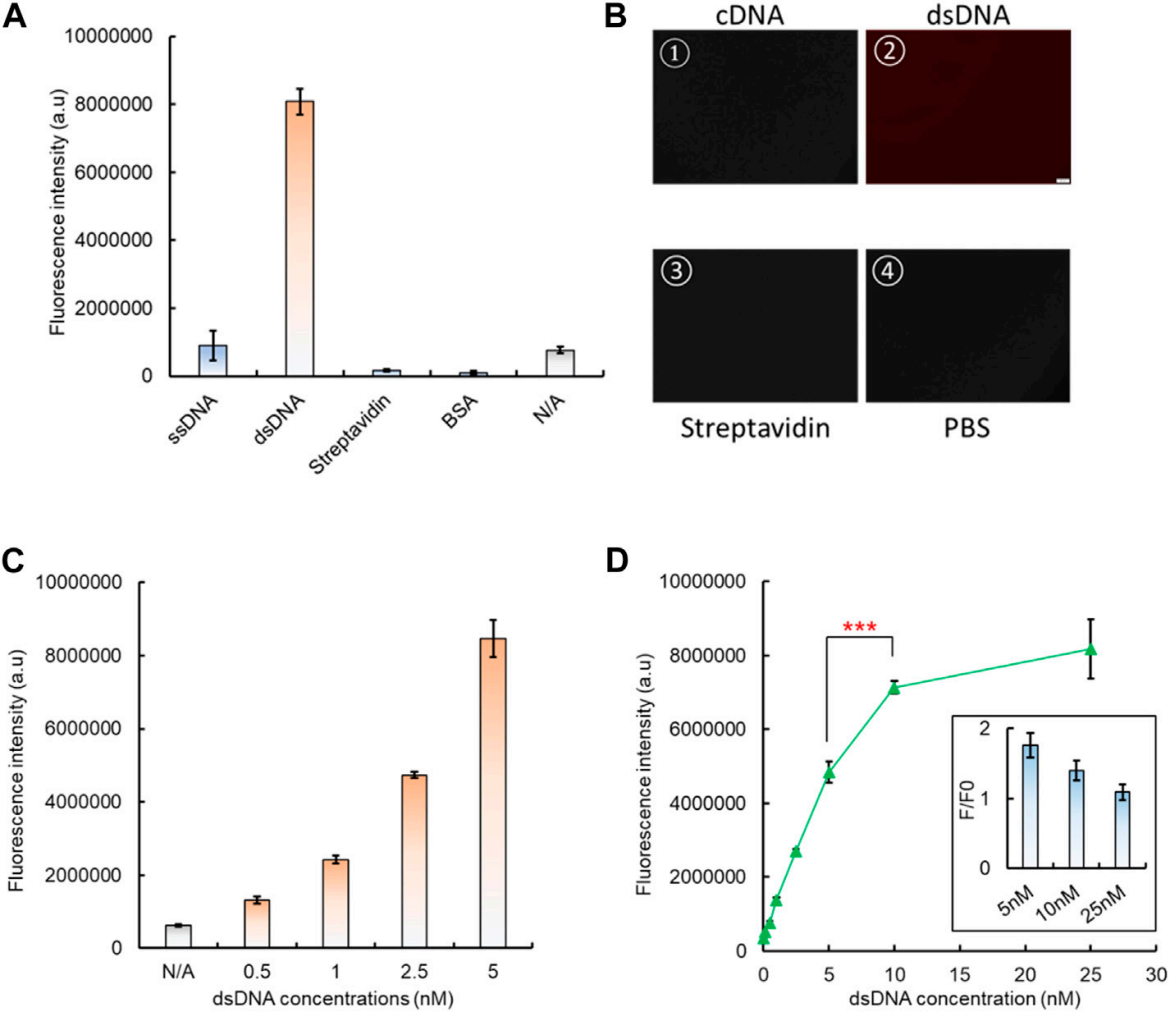
Fixation of the dsDNA biosensing module onto a 96-well plate. **(A)** Basic controls to demonstrate the successful fixation of dsDNA onto a high-binding 96-well plate. **(B)** Verification of dsDNA fixation by fluorescent microscopy. **(C)** Fluorescent signal increase shown by increased dsDNA fixation onto a 96-well plate. **(D)** Optimization of the dsDNA concentration to form the dsDNA biosensing interface, along with the consideration of its response to the analyte, which is related to the dsDNA unwinding and signal generation. The insert presents the change of the fluorescence ratio between the dsDNA response to its analyte (F) and the background signal (F0). This served as the indicator to evaluate the dsDNA biosensing function.

To integrate the dsDNA biosensor with CRISPR/Cas12a to form a comprehensive system, it needs to be attached onto a solid interface, so the released aptamer–analyte complex can be removed. Here, a standard high-binding 96-well plate was applied as the substrate for the aptamer-based dsDNA biosensing module attachment. After the 96-well surface was coated with 100 µL 4 μg/mL streptavidin, the premade dsDNA biosensor was added, with biotin-labeled cDNA and the Texas Red-labeled aptamer. After BSA blocking and PBS washing, only the well surface with both streptavidin and dsDNA showed a significantly higher fluorescence signal, which indicated the successful coating of the dsDNA biosensor onto the 96-well plate, and formation of the biosensing interface (Figure 4A). A similar increased fluorescence signal was also observed by fluorescence microscopy. After PBS washing, only the addition of dsDNA leads to a bright red fluorescence signal from the well surface (Figure 4B), which further verifies the attachment of our dsDNA biosensor onto the 96-well plate surface. By increasing the concentration of the aptamer–cDNA dsDNA from 0.5 nM to 5 nM, the fluorescence intensity also increased (Figure 4C), and such an increasing trend can also be directly observed from the solid surface of the high-binding plate by confocal microscopy when the dsDNA concentration increased from 0 nM to 100 nM (Supplementary Figure S10).

The optimal dsDNA concentration to form the biosensing interface was also investigated. The results have shown that the fluorescence intensity continued to increase with concentrations from 0.5 nM, and it saturated at 10 nM (Figure 4D). However, when the fluorescence-quenched dsDNA biosensor was used to measure the presence of the analyte, we found that the biosensing interface formed with 5 nM dsDNA generated the highest rate of fluorescence intensity change compared to 10 nM (Figure 4D). Therefore, the 5 nM concentration was selected for dsDNA biosensing interface fabrication in subsequent tests.

### 3.5 Integrated CAMERA biosensing system for IFN-γ detection

**FIGURE 5:**
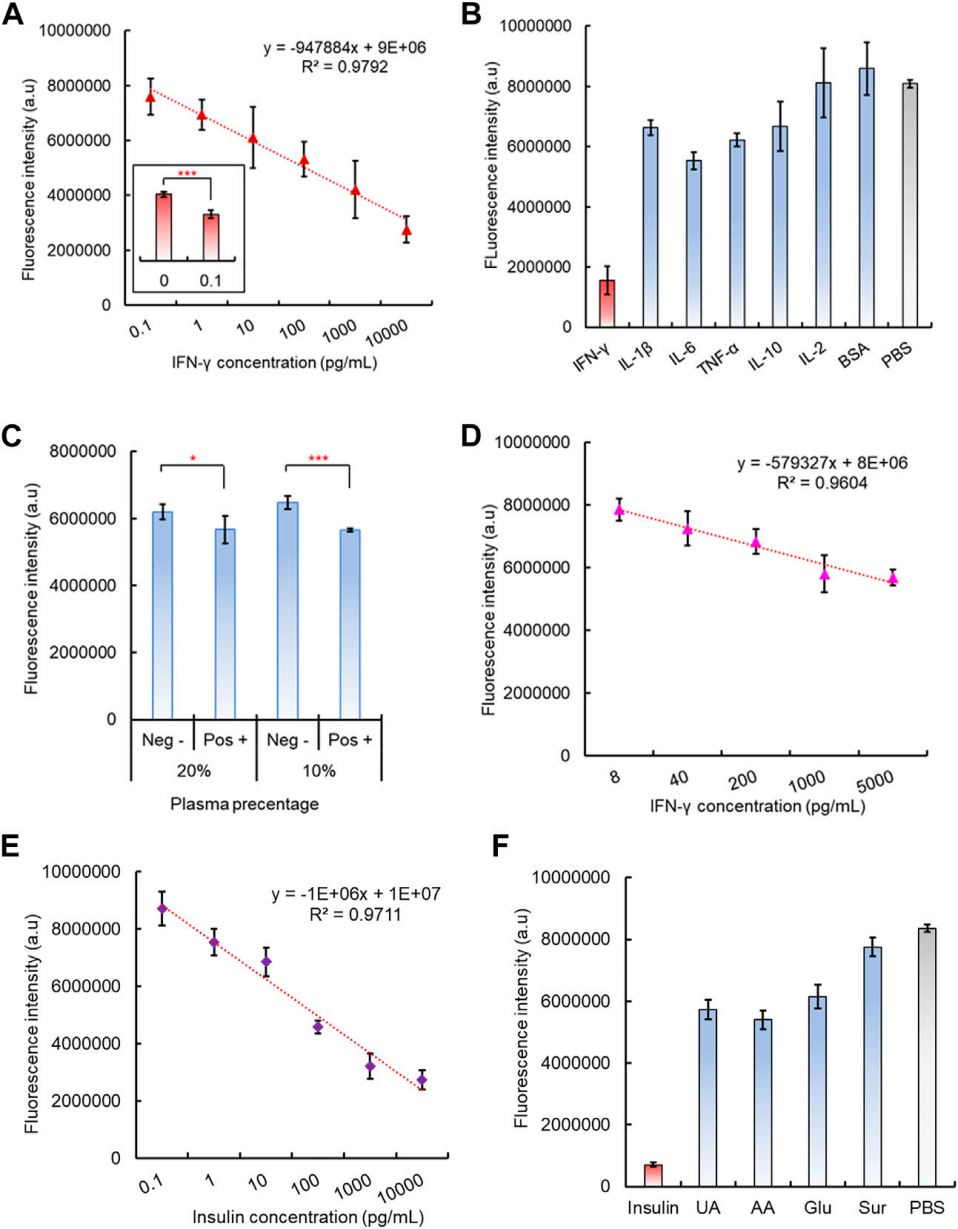
Performance of the integrated CAMERA system for IFN-γ detection. **(A)** Linear correlation of CAMERA for IFN-γ detection. **(B)** Specificity test of CAMERA for IFN-γ detection. **(C)** Feasibility of CAMERA in diluted plasma samples. **(D)** Linear correlation of CAMERA for detecting IFN-γ from 10-times diluted plasma. **(E)** Linear correlation of CAMERA for insulin detection. **(F)** Specificity test for insulin detection.

After preparation of the 96-well plate dsDNA biosensing interface and its corresponding CRISPR/Cas12a reaction mixture, the capability of the integrated CAMERA system to detect cytokine IFN-γ was evaluated. The results showed that the final fluorescence intensity decreased with the IFN-γ concentrations increased from 100 fg/mL to 10 ng/mL, and a negative linear correlation was observed with *R* ^2^ = 0.9792 (Figure 5A). The statistical analysis indicated a significantly different fluorescence signal between IFN-γ concentration 0 (PBS solution) and 100 fg/mL, which indicated the tested detection limit to be 100 fg/mL (Figure 5A). The system specificity has also been tested by introducing several interference cytokines and small proteins, including IL-β, IL-6, TNF-a, IL-10, and IL-2 and BSA, and PBS solution has been used as control. Although some interference molecules, such as IL-1β, IL-6, and TNF-a, also exhibited a noticeable decrease in fluorescence intensity compared to the PBS control, only the presence of IFN-γ showed a significantly decreased fluorescence intensity (Figure 5B).

To further test the potential feasibility of our CAMERA system in dealing with clinical samples, IFN-γ spiked plasma samples have also been used, diluted in PBS at 0%, 20%, 40%, 60%, 80%, and 90%, respectively. The results indicated that our CAMERA system has not been able to respond to the IFN-γ spiked plasma samples unless the plasma has been diluted to 20% (5X dilution) or 10% (10X dilution) in PBS (Figure 5C, Supplementary Figure S11). Afterward, different concentrations of IFN-γ were spiked into 10X diluted plasma samples, and a negative linear correlation was observed from 8 pg/mL to 5 ng/mL (Figure 5B). This indicated that the actual sensitivity for testing the plasma sample is around 8 pg/mL, which is slightly higher than the normal range in human samples (Caldas et al., 2005). Therefore, although CAMERA has demonstrated its capability to work with more complex biological samples, compared to testing IFN-γ in PBS samples, plasma samples were found to be more suitable for testing the target analyte at higher concentrations.

### 3.6 Demonstration of CAMERA universality

To investigate the potential of our CAMERA system in applying to a number of alternative analytes, another type of small protein, insulin (hormone, 5.8kD), was also investigated. Similarly, as in the case of IFN-γ, insulin aptamers were used to form the aptamer-based dsDNA biosensors with corresponding cDNA oligos. Accordingly, CRISPR/Cas12a RNP used a modified gRNA sequence, which was complimentary to the insulin aptamer. Both the results of fluorescence quenching and gel electrophoresis assays verified the successful formation of the expected dsDNA structure (Supplementary Figures S12, S13). In addition, the response of the formed insulin dsDNA biosensor response to its target analyte was tested. In the presence of insulin, the dsDNA biosensor has shown a significant fluorescence signal recovery (Supplementary Figure S14). In addition, the aptamer-triggered CRISPR/Cas12a collateral cleavage activity was verified for the insulin aptamer with CRISPR/Cas12a RNPs with corresponding gRNA sequences. The data indicated that similar Cas12a triggering patterns have been detected, and the amplified signal intensity was more pronounced with increasing reaction time (Supplementary Figure S15). Subsequently, the complete CAMERA protocol was performed for insulin detection. The results have shown that our CAMERA is capable of quantitative detection of insulin with the same 100 fg/mL sensitivity and linear range from 100 fg/mL to 10 ng/mL (Figure 5E), which is compatible with the results for IFN-γ detection. In addition, satisfactory system specificity was also observed for this additionally tested analyte (Figure 5F).

## 4 Discussion

Recent progresses in CRISPR/Cas-based biosensor development have led to impressive performance in sensitivity, specificity, and speed for nucleic acid detection (Zuo et al., 2017; Chertow, 2018). More importantly, with the combination of other biosensing elements, CRISPR/Cas RNP exhibited excellent compatibility with other biosensing elements in development of different biosensing systems for targeting a variety of analytes other than nucleic acid (Zhang et al., 2021). By directly combining aptamer-based target recognition with the collateral cleavage of CRISPR/Cas12a for signal amplification, we have successfully developed a simple but universal biosensing platform for non-nucleic acid detection. The CAMERA system is composed of two simple modules for detection, the aptamer-based dsDNA biosensing module for target recognition and binding—which leads to the separation of its dsDNA structure—and the CRISPR/Cas12a-based fluorescence signal amplification module corresponding with the aptamer nucleic acid sequence for amplified signal generation. Eventually, the integrated system demonstrated a quantitative negative correlation between the amplified fluorescence signals and the concentration of target analytes. It has been demonstrated to detect small proteins, such as IFN-γ and insulin, and shows potential to be applied to a wider range of various analytes with properly selected aptamers (Song et al., 2008; Zhou et al., 2014).

Two critical elements have ensured the high sensitivity and specificity of our CAMERA system. First is the successful attachment of the dsDNA biosensor onto the solid surface, which allows us to easily separate and remove the unwanted analyte-bound aptamer from the reaction mixture in contrast to earlier studies (Dai et al., 2019; Liang et al., 2019). This separation eliminates the accidental activation of CRISPR/Cas12a signal amplification, which is unrelated to the analyte signal, and it leads, in our case, to a reduced unspecific background. Second, the programmable nuclease activity of the Cas12a protein, which has single-nucleotide specificity, allows us to specifically control the activation of signal amplification through modifying its guide RNA sequence (Chen et al., 2018).

More importantly, the whole biosensing system is established based on a standard 96-well plate with similar experimental procedures to conventional ELISA. This makes our CAMERA system a user-friendly replacement for antibody-based ELISA with no need for additional instruments or training. Compared to commercialized ELISA kits, which generally have tested sensitivities from tens to hundreds pg/mL (Li et al., 2021c; Li et al., 2022), CAMERA has shown an improved performance in sensitivity, while with a similar level of specificity and shorter detection time. In dealing with ≥10 times diluted plasma samples, our CAMERA system also exhibits a wider linear range (2–3 logs) in comparison to a conventional ELISA kit, which can be important in detecting analytes with highly diverse concentrations in clinical samples, such as cytokines in the human body (Iturriaga, 2014) or toxins in the environment (Mauro and Koudelka, 2011). Additionally, we used aptamers for target recognition. They have small molecular weight and a simple nucleic acid structure for easy manipulation and synthesis (Mehlhorn et al., 2018; Munzar et al., 2019), and they can tolerate dramatic temperature variations while still retaining their designed target affinity when they are returned back to their working temperature (Munzar et al., 2019). In addition, aptamers are commonly chemically synthesized. Therefore, CAMERA offers a more cost-efficient replacement approach in comparing the antibody for target recognition. It can be easily manipulated to match various targets, with lower cost and faster time for system development.

The CAMERA developed in this study presents a novel strategy to apply aptamer-based biosensors by integrating with CRISPR/Cas12 biotechnology, achieving higher sensitivity and a wider linear range for target detection. It is simple, universal, versatile, and compatible with current ELISA measurement setups, such as plate readers. Moreover, as aptamers are generally not temperature-sensitive and can work at various temperature ranges, such as 37°C (Munzar et al., 2019), it shows potential to be adapted for *in vivo* diagnostic applications, e.g., using an implantable device for IL-1β detection, as reported by Deng et al. (2020) Meanwhile, as both the aptamer and Cas12a proteins retain their functions on paper-based substrates, the CAMERA system shows potential to be transferred to microfluidic paper-based analytical devices, such as paper lateral flow assay test strips, to realize the POC diagnostics. Although applying the aptamer may raise potential limitations on its availability to different analytes and sensitivity to nuclease contaminations, which can be relieved with the further expanded aptamer pool and additional nucleic acid modification techniques, we expect that the CAMERA method will inspire the use of CRISPR/Cas biosensing technology in combining with other existing biosensing components for novel biosensor development.

## Data Availability

The raw data supporting the conclusion of this article will be made available by the authors, without undue reservation.
