# Supplementary material for: Universal CRISPR/Cas12a-associated aptasensor suitable for rapid detection of small proteins with a plate reader
Source: Front Bioeng Biotechnol. 2023 Jun 2;11:1201175. doi: 10.3389/fbioe.2023.1201175 (PMC10272437; doi:10.3389/fbioe.2023.1201175)
Supplement: Supplementary file 1 [file DataSheet1.docx]

**Universal CRISPR/Cas12a-associated aptasensor suitable for rapid detection of small proteins with plate reader**

Yi Li ^a,b*^, Linyang Liu ^a,b^, Laicong Qiao ^a,b^, Fei Deng ^a,b^

^a^ Graduate School of Biomedical Engineering, Faculty of Engineering, University of New South Wales, Sydney 2052, Australia

^b^ ARC Centre of Excellence for Nanoscale Biophotonics, University of New South Wales, Sydney 2052, Australia

* Correspondence: [yi.li6@unsw.edu.au](mailto:yi.li6@unsw.edu.au)

###

### Supplementary figures


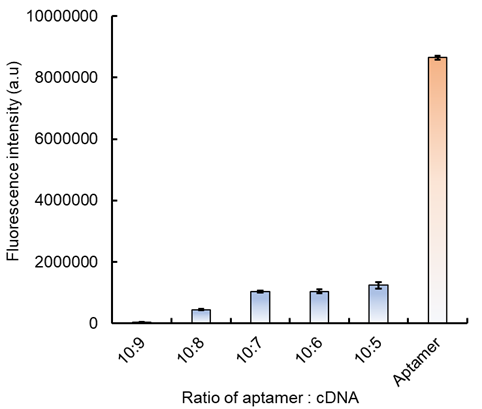


**Figure S1** Quenching efficiency for different aptamer to cDNA ratio.


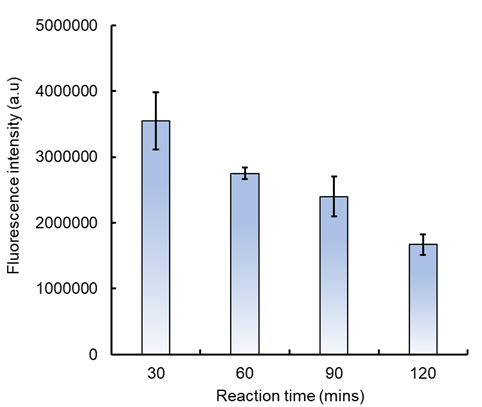

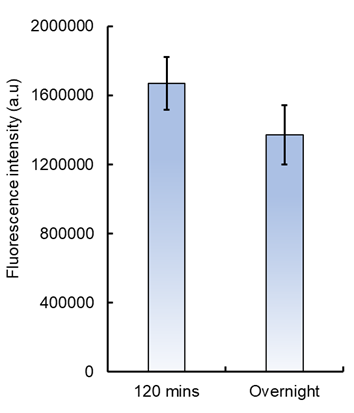


**Figure S2** Verification the status change of dsDNA formation (quenching) during different time. No statistical different between 60 to 90, or 90 to 120. Also, the overnight fluorescence intensity decrease is not significant compared to 120 mins.


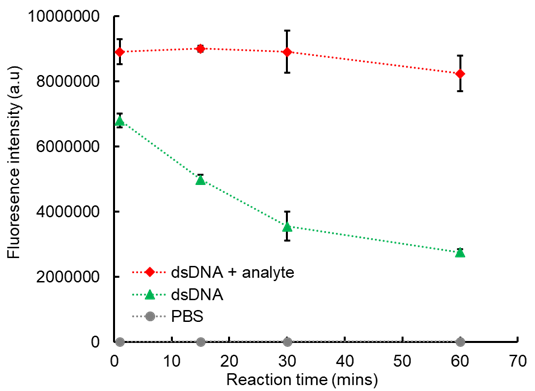

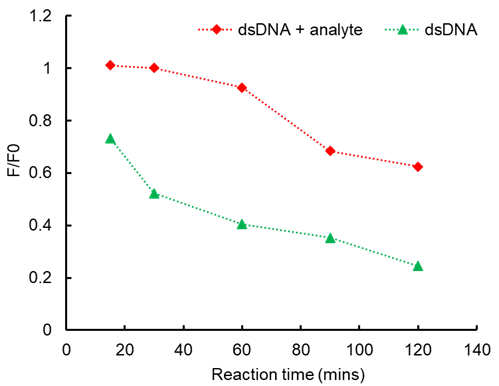


**Figure S3** Competitive binding test between analyte and cDNA to the aptamer. dsDNA indicated the mixture of aptamer and cDNA. Data indicated the aptamer has higher affinity to analyte compared to its cDNA. The fluorescence signal intensity shows no significant difference between 1 min to 60 mins; this also indicates that the decay of fluorescence signal due to quenching is negligible.


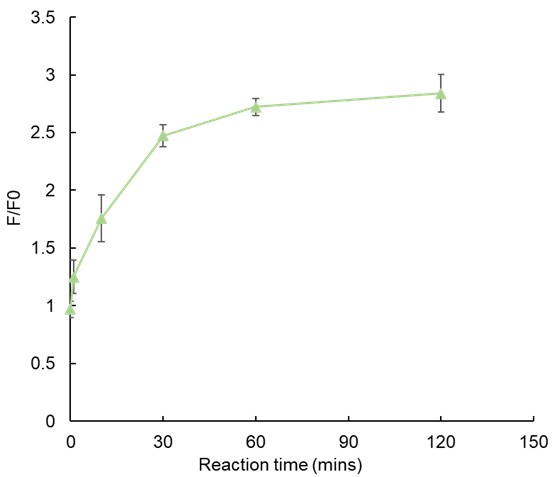


**Figure S4** The dsDNA unwinding test for dsDNA biosensor in the presence of target analyte. Data show the correlation between the reaction time and fluorescence intensity changes. The saturated fluorescent signal intensity at 60 mins indicated that the optimal reaction time for dsDNA biosensor to response to its analyte is 60 mins.


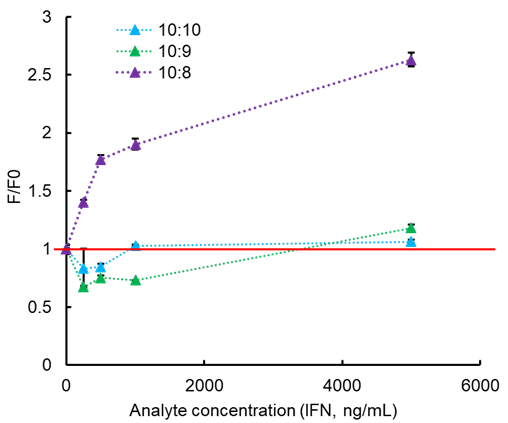

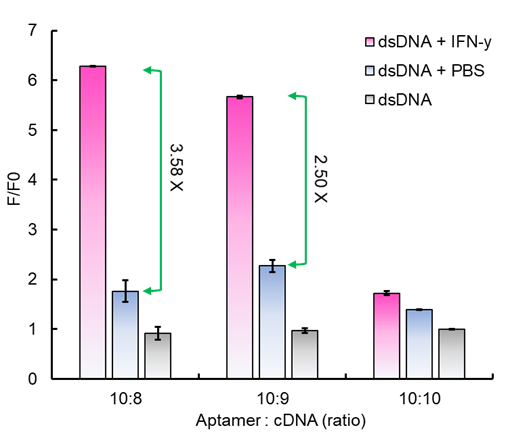


**Figure S5** The change of dsDNA unwinding level under different aptamer to cDNA ratios.


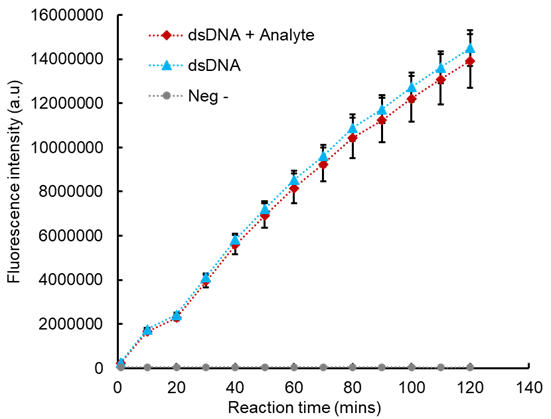


**Figure S6** Investigate the Cas12a activation efficiency changes with the presence of target analyte. Data indicated that the presence of analyte has no significant effect on CRISPR/Cas12a signal amplification module.


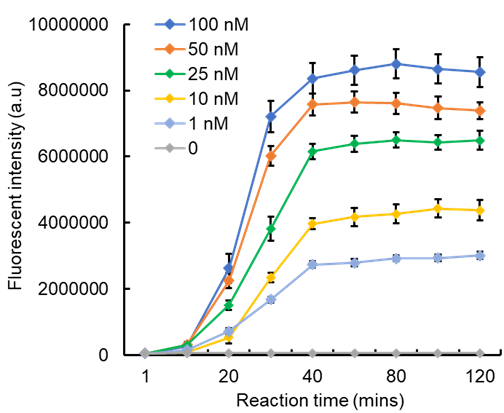


**Figure S7** The Cas12a collateral cleavage intensity changes with different gRNA concentrations.


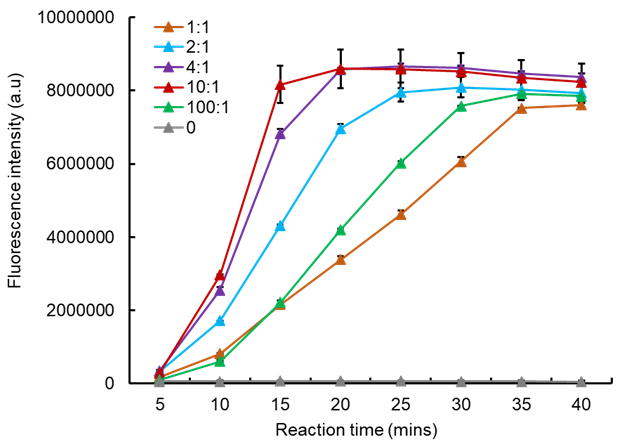


**Figure S8** Optimization of the gRNA : Cas12a ratio for signal amplification efficiency.


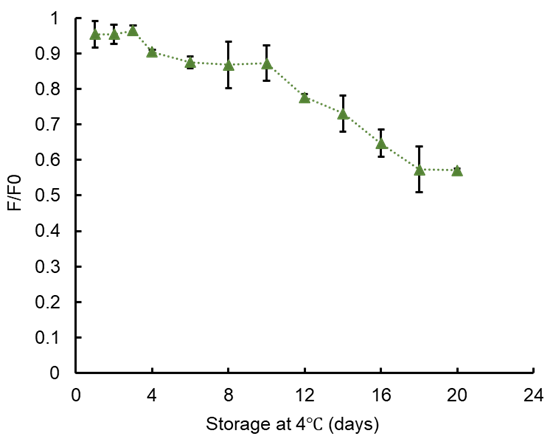


**Figure S9** Investigation of CRISPR/Cas12a RNP stability within 1X NEB 2.1 buffer.


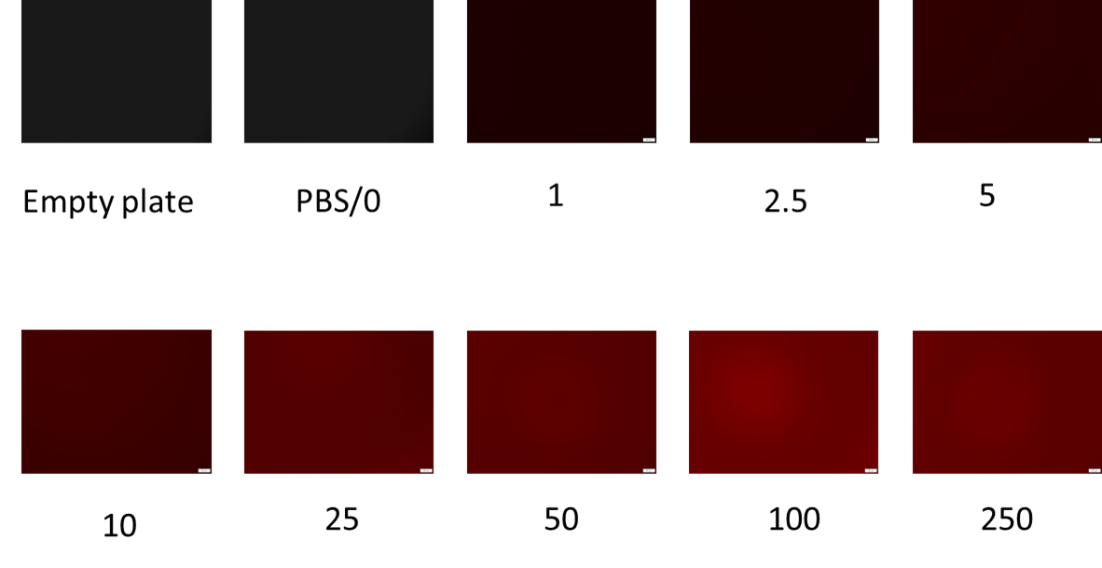


**Figure S10** Demonstration of the fixation of dsDNA biosensor onto the solid surface of 96-well high-binding plate. The dsDNA concentration for surface coating from 1 nM to 250 nM.


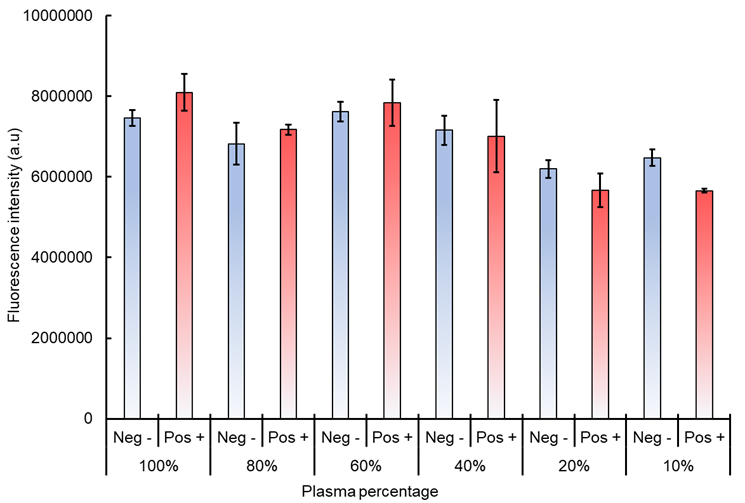


**Figure S11** CAMERA response to IFN-γ in Plasma samples with different percentage of plasma in PBS buffer.


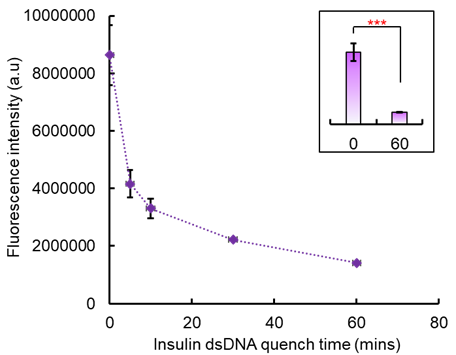


**Figure S12** Fluorescence quenching test to verify the formation of dsDNA for insulin aptamer and its cDNA.


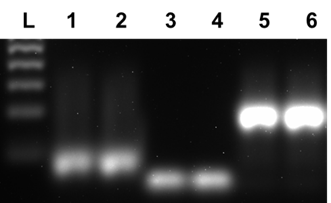


**Figure S13** Verifying the formation of dsDNA with insulin aptamer and cDNA by EMSA. Lines: 1, 2 = insulin aptamer; 3, 4 = cDNA; 5, 6 = dDNA.


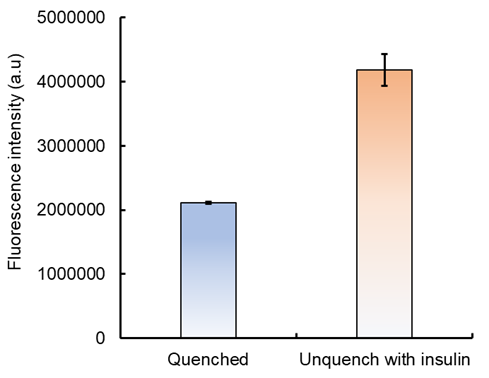


**Figure S14** Demonstration of the insulin dsDNA biosensor by analyte induced fluorescent signal recovery test (t.test=0.0078, Insulin concentration = 1.25 µg/mL).


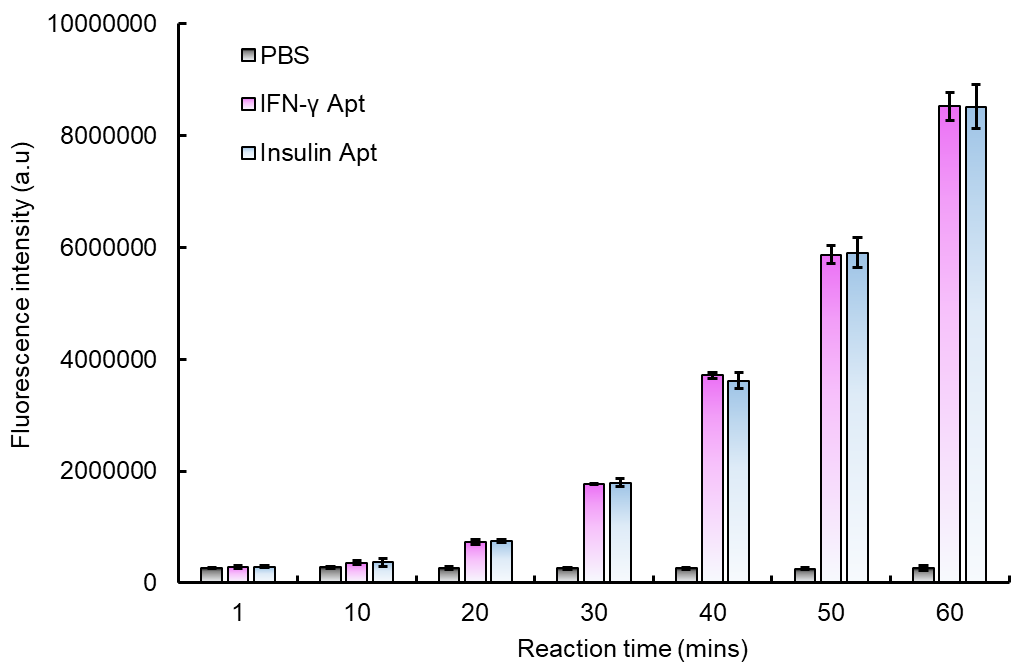


**Figure S15** Demonstration of insulin aptamer triggered Cas12a collateral cleavage for fluorescent signal amplification.

### Supplementary tables

**Table S1. Synthesized oligos for CAMERA development**

| Name | Oligo type | Sequence 5’-3’ | Length (nt) | Modification |
| --- | --- | --- | --- | --- |
| Insulin aptamer gRNA | ssRNA | UAA UUU CUA CUA AGU GUA GAU GAC ACC CUA CCA ACC CCC CCC | 42 | N/A |
| Insulin aptamer 1 | ssDNA | GGT GGT GGG GGG GGT TGG TAG GGT GTC TTC | 30 | 5’-BHQ2 3’-biotin; |
| Insulin aptamer 2 | ssDNA | GGT GGT GGG GGG GGT TGG TAG GGT GTC TTC | 30 | N/A |
| Insulin cDNA 1 | ssDNA | GAA GAC ACC CTA CCA ACC CCC CCC ACC ACC | 30 | 3’-Texas red |
| Insulin cDNA 2 | ssDNA | GAA GAC ATT TTT TCC AAC CCC | 21 | 3’-biotin |
| IFN-y aptamer gRNA | ssRNA | UAA UUU CUA CUA AGU GUA GAU ACA ACA CCC AAC ACA ACC AAC CCC | 45 | N/A |
| IFN-y aptamer 1 | ssDNA | GGG GTT GGT TGT GTT GGG TGT TGT GT | 26 | 5’-BHQ2 3’-biotin; |
| IFN-y aptamer 2 | ssDNA | GGG GTT GGT TGT GTT GGG TGT TGT GT | 26 | N/A |
| IFN-y cDNA 1 | ssDNA | GAA GAC ATT TTT TCC AAC CCC | 21 | 3’-Texas red |
| IFN-y cDNA 2 | ssDNA | GAA GAC ATT TTT TCC AAC CCC | 21 | 3’-biotin |
| ssDNA reporter | ssDNA | TTATT | 5 | 5’-Texas Red; 3’-BHQ2 |
